# Supplementary material for: Scoping Review of Peer-Led Support for People Bereaved by Suicide
Source: Int J Environ Res Public Health. 2022 Mar 15;19(6):3485. doi: 10.3390/ijerph19063485 (PMC8954346; doi:10.3390/ijerph19063485)
Supplement: Supplementary file 1 [file ijerph-19-03485-s001.zip › Supplementary Table 1.pdf]

Supplementary Table S1: Sample MEDLINE Search.

#	Query	Results
S10	S3 AND S6 AND S9	2,461
S9	S7 OR S8	101,490
S8	(MH "Suicide, Completed") OR (MH "Suicide")	40,789
S7	"self killing" OR suicid* OR "kill* themsel*" OR "kill* oneself*" OR self-killing OR "taking their own life" OR "ending their life"	101,490
S6	S4 OR S5	1,147,636
S5	(MH "Bereavement+") OR (MH "Disenfranchised Grief")	13,748
S4	(bereave* OR grie* OR loss* OR mourn* OR "death of a loved one*" OR "loss of a loved one*" OR stigma* OR guilt* OR shame* OR "disenfranchised grie*") N3 (famil* OR relative* OR spouse* OR parent* OR husband* OR wife* OR mother* OR father* OR sibling* OR grandparent* OR grandmother* OR grandfather* OR widow* OR child* OR acquaintance* OR friend* OR student*) OR bereave* OR grie* OR loss* OR mourn*	1,147,636
S3	S1 OR S2	6,737,034
S2	(MH "Self-Help Groups") OR (MH "Social Support+") OR (MH "Peer Group+") OR (MH "Peer Influence")	99,857
S1	AB (Peer* OR group* OR collect* OR unif* OR mutual* OR retreat* OR Restorativ* OR shar* OR "social support*" OR self-help* OR "self help*" OR psychosocial* OR psycho-social* OR postvention* OR "survivor* support*" OR survivor-support* OR "para professional support*" OR "para-professional support" OR "paraprofessional support*" OR prevent*) OR TI (Peer* OR group* OR collect* OR unif* OR mutual* OR retreat* OR Restorativ* OR shar* OR "social support*" OR self-help* OR "self help*" OR psychosocial* OR psycho-social* OR postvention* OR "survivor* support*" OR survivor-support* OR "para professional support*" OR "para-professional support" OR "paraprofessional support*" OR prevent*)	6,701,666
