# Supplementary material for: Scoping Review of Peer-Led Support for People Bereaved by Suicide
Source: Int J Environ Res Public Health. 2022 Mar 15;19(6):3485. doi: 10.3390/ijerph19063485 (PMC8954346; doi:10.3390/ijerph19063485)
Supplement: Supplementary file 1 [file ijerph-19-03485-s001.zip › Supplementary Table 2.pdf]

Supplementary Table S2: Study Characteristics.

Author (Location)	Type of peer intervention	Aims and objectives of the evaluation	Study type	Data collection method and data analysis	Study sample	Ethics approval and consent	[1]Quality assessment
Hopmeyer & Werk, 1994 (Canada)	An open ended continuous group for any family member who has lost someone to suicide. Facilitated by a peer without professional training but a professional (social worker) attended most meetings as a consultant/resource person. Group size 5-12 individuals.	To evaluate and improve the group service offered to five bereavement groups	Survey	Closed questions on people's social network, reasons for attending the group, satisfaction/dissatisfaction with the group and ideal group format.<br>Open ended questions<br>Data analysis: Desriptive statistics	Age, gender, relationship to the deceased and duration of time since loss: not reported	Neither mentioned	Weak
Feigelman et al. 2008 (USA)	An online internet support group known as Parents of Suicide (POS). Group started by a bereaved parent.<br>Members were free to carry on separate dialogues between one another and there were guidance given to members by the facilitatoron how to frame their submissions to attract the widest response from other support group	To investigate how suicide survivors participate in Internet support groups	Survey	Survey -2 stages.<br>Stage 1: Used the internet support group utilization survey.<br>Stage 2: For those who agreed a more detailed survey followed which used -the Complex Grief Scale; the Impact of Events Scale; the Family/Social Strain Scale, the Family Unhelpful	N= 116 in stage 1 and 540 in stage 2. Age, gender, relationship to the deceased and duration of time since loss of total sample: not reported	Neither mentioned	Moderate

[1] The CASP consists of ten items (clarity of aims, appropriateness of design methodology and recruitment, rigor of data collection and analysis, ethical issues, relationship between researcher and participants, clarity, adequacy, and relevance of findings) which are scored on a scale of 0-1 and summed to determine a total CASP score. The maximum score possible score of 10, with scores 0-4.5 points considered low/weak, score of 5-7.5 points were medium/moderate quality and a scores of 8-10 points was considered high/strong quality. The Effective Public Health Practice Project Quality Assessment tool (Thomas et al 2004) was used for studies using quantitative design. This tool also had 10 domains (selection bias, type of design, confounders, blinding, withdrawals, validity and reliability of data collection, sampling, type of data, validity of evaluation instrument, intervention integrity, appropriateness of data analysis). Each domain was rated using the categories of strong, moderate, or weak. Those awarded a strong rating have no weak ratings, with those given a moderate or weak rating having one weak rating or two or more WEAK ratings, respectively.

Author (Location)	Type of peer intervention	Aims and objectives of the evaluation	Study type	Data collection method and data analysis	Study sample	Ethics approval and consent	[1]Quality assessment
	participants.			Response Scale, the social unhelpful response scale, plus question on depression and suicide ideation. Data analysis: Desriptive and inferential statistics			
Feigelman & Feigelman, 2008 (USA)	A face to face open-ended support group which was . a two-hour monthly meeting . Meeting began with informal social period followed by , an introduction by the facilitator who encouraged group members to briefly introducing themselves, stating their name, relationship to the deceases, and the self-killing method employed. New members were offered the option of passing over their turn if they felt unable to share at this time. After the initial go-around, the facilitator opened the meeting to those wishing to make general remarks and to respond, non-judgmentally, to each other. Periodically the facilitator redirected the flow of communication and assured new members opportunities to speak if they wished to do so. At the close of the meetings, the facilitator led	To explore how peer-led bereavement support groups offer participants meaningful help with their grief and loss experiences	Qualitative	Participant observation of one group over a 4-year period. Survey of bereavement experiences (findings not reported in the paper). Data analysis: Schulman's dynamics of mutual aid theory	N= 15-35 people in a suicide (peer-led) support group. Age, gender, and duration of time since loss of total sample: not reported ed<br><br>Attendees held a variety of relationships to deceased: parents, children, partners, spouses, siblings, other relatives, and friends	Ethcical approval not mentioned. Consent not sought.	Weak

Author (Location)	Type of peer intervention	Aims and objectives of the evaluation	Study type	Data collection method and data analysis	Study sample	Ethics approval and consent	[1]Quality assessment
	the group in a reading of the serenity prayer, which was then followed by another informal socializing period where members shared refreshments						
Barlow et al. 2010 (Canada)	A face-to-face peer supporter-client dyad (both bereaved by suicide) was matched according to gender, type of loss, age, and relationship to the deceased. Regularly, meetings for 4 months. Some dyads, after meeting several times, chose to continue meetings via phone as it was deemed easier logistically.<br>The peer supporters were trained volunteers and partook in monthly debriefing and educational sessions.	To assess the impact of peer support program on participants' level of grief and heali ng, the ability of the program to meet participants' personal goals, the helpfulness, and the level of comfort at meetings.	Mixed methods design	Interviews and surveys.<br>Survey: Pre and post Hogan Grief Reaction Checklist, plus open and closed questions.<br>Data analysis: Inferential statistics and content analysis	N=16 (9 peer supporters and 7 client participants)<br>**Age:**<br>Range from 26-66 years<br>Average age of 46.9<br>**Gender:**<br>13 women<br>3 men<br>**Relationship to the deceased:**<br>Wives, husband, fathers, sons, sisters, and common-law husbands<br>**Duration of time since suicide:**<br>Range from 6 weeks to 20 years<br>12 report death within the past 5 years	Yes	Weak
Feigelman & Feigelman, 2011 (USA)	See Feigelman & Feigelman, 2008.	To examine survivors needs, including reasons for leaving peer support group for people	Qualitative	Telephone interviews<br>Data analysis approach not stated	**N=** 24 participants who withdrew from group<br>**Age:** not reported<br>**Relationship to deceased:** parents, children, siblings, friends, relatives, spouses or partners, other<br>**Duration of time since suicide:**	Ethical approval not mentioned. Consent sought for interviews but not for participant	Moderate

Author (Location)	Type of peer intervention	Aims and objectives of the evaluation	Study type	Data collection method and data analysis	Study sample	Ethics approval and consent	[1]Quality assessment
		bereaved by suicide.			1/3 experienced the loss to suicide within 2 years<br>Majority experienced loss 5+ years previously	observation.	
Marijke Schotanus-Dijktra et al. 2013 (Belgium and Netherlands)	Online support group. Facilitated by volunteer with professional back up.	To determine what is communicated by participants in online support groups for the bereaved by suicide.	Qualitative	Analysis of a large sample of messages in two online forums<br>Data analysis: Content Analysis	N = 164/165 participants 'most likely Dutch or Belgian'<br>**Age:**<br>Mean age of 32 years (only identified in 46 members)<br>**Gender:**<br>70% women; 8% men; 22% unknown<br>**Relationship to deceased:**<br>24% parent; 21% partner;19% sibling; 9% child; 6% friend; 11% other;5% unknown<br>5% experienced the loss of more than one person<br>**Duration of time since suicide:**<br>22% 0-3 months; 13% 3-6 months<br>12% 6-12 months; 25% 1-5 years<br>20% > 5 years; 8% unknown	Ethical approval not mentioned. Authors state that consent was not necessary	Strong
Bailey et al. 2017 (UK, USA, Canada and Australia)	Online internet forums (n=9) and Facebook groups (n=8) specific for people bereaved by suicide. Groups appeared to be managed by suicide bereavement organizations or by other individuals, but who these were was not clear.	To explore what internet forums are used by those bereaved by suicide, including perceived	Survey	Online survey using the Kessler psychological distress scale, forum use, help-seeking questionnaire (GHSQ & AHSQ), and questions on perceived benefits and limitations of forum use.	N=222<br><br>**Age:**<br>Range from 17-81 Mean: 52.3<br>**Gender:**<br>94.6% female<br>**Relationship to bereaved**<br>60.8% child; 28.4% friend;	Yes	Moderate

Author (Location)	Type of peer intervention	Aims and objectives of the evaluation	Study type	Data collection method and data analysis	Study sample	Ethics approval and consent	[1]Quality assessment
		benefits and limitations of use		To identify online groups two systematic searches were conducted using: (a) web-based search engines including Google, Bing, Yahoo, and Ask); (b) Facebook's internal search tool.<br>Data analysis: Descriptive statistics	26.6% acquaintance; 13.5% partners; 11.7% siblings; 8.6% parent; 5.9% colleagues; 28.8% other<br>59.5% knew more than 1 person who had died by suicide.<br>**Duration of time since suicide:** 14.9% bereaved 6-12 months ago; 49.5% bereaved between 1-5 years ago;<br>34.7% bereaved more than 5 years ago		
Silvén Hagström, 2017 (Sweden)	An internet blog with website editors responded to posts. The website was created by two young women who both lost a younger brother to suicide: one after a long struggle with mental illness and another from a sudden and unexpected suicide).	To investigate how an internet community can offer social support to people bereaved by suicide with grieving.	Qualitative	Analysis of communication between the website editors and participants on a Swedish website for suicide bereaved over a five-year period.<br>Data analysis: Narrative analysis	610 chat messages<br>Age, gender, relationship to the deceased and duration of time since loss of people in the internet community not reported	Neither mentioned	Strong
Ali & Lucock, 2020 (UK)	Face to face meetings of around two hours typically once a month. Volunteer-led group.	To develop a theory on how an individual internalises and actively constructs meaning through their	Qualitative	Semi-structured in-depth interviews and a focus group<br>Data analysis: Constructivist Grounded Theory	**N=** 22<br>**Age:** range from 23-67 years<br>**Gender:**<br>16 female; 6 male<br>**Relationship to the deceased:**<br>12 parents; 3 husbands; 2 wives; 2 siblings; 2 extended family members	Yes	Moderate

Author (Location)	Type of peer intervention	Aims and objectives of the evaluation	Study type	Data collection method and data analysis	Study sample	Ethics approval and consent	[1]Quality assessment
		social relationships and interactions with others who were attending a Survivors of Bereavement by Suicide (SOBS) support group			1 child<br>**Duration of time since suicide:** Varied from 3 months-19 years; Average of 3 years		
Tosini & Fraccaro, 2020 (Italy)	The group was facilitated by a voluntary facilitator (also a survivor) from a network of support groups.<br>The suicide-specific group which was observed met weekly for approx. 90 mins.	Identify how the survivors' involvement in mutual aid groups influenced their grieving process, with the main focus on the stories of participants in a suicide specific group	Qualitative	Semi-structured interview and analysis of field notes from participant observation of 47 meetings of the suicide-specific group<br>Data analysis: Thematic analysis	**N=**25<br>**Gender:**<br>22 women<br>3 men<br>**Age:** Range from 30-69<br>**Relationship to deceased:**<br>10 female partners; 6 mothers; 1 husband; 1 father; 1 daughter; 1 sister; 2 nieces; 1 uncle; 1 cousin;<br>1 sister-in-law.<br>**Civic Status:**<br>18 married; 1 divorced; 3 widowed;<br>3 single	Yes	Moderate
