# Supplementary material for: Scoping Review of Peer-Led Support for People Bereaved by Suicide
Source: Int J Environ Res Public Health. 2022 Mar 15;19(6):3485. doi: 10.3390/ijerph19063485 (PMC8954346; doi:10.3390/ijerph19063485)
Supplement: Supplementary file 1 [file ijerph-19-03485-s001.zip › Supplementary Table 3.pdf]

Table S3: Findings

Article (Location)	Identify motivation/ impact/outcomes/benefits of peer interventions	Elements of peer intervention that enhanced outcomes	Elements of peer intervention that hindered outcomes
Hopmeyer & Werk (Canada)	**MOTIVATION:**<br>Reasons for joining group -ranked as 'very important':<br>Share similar experience (85%)<br>Gain comfort and reassurance (76%)<br>Learn new coping skills (76%)<br>Share what I learned (45%)<br>There was no one else (41%)<br>I was depressed (41%)<br>Get advice (41%)<br>Help others (31%)<br>Get information (30%)<br>I was desperate (21%)<br>Make new friends (10%)<br>Curiosity (7%)<br><br>**IMPACT:**<br>Most valuable gains from the group:<br>1) not alone<br>Quote: *"The contact with another person in the group who had experienced things very similar to me gave me the feeling that I am not alone"* (p253)<br>Quote: *"liked the sense of being in the same situation as others – the all-in-the-same boat feeling"* p252<br>2) not the only one<br>3) someone understands<br>4) Shared my grief<br>5) not alone in my pain<br>6) others have similar tragedies (p252)<br><br>A feeling of acceptance and being around people who understand – helped not to		

	reduce self-blame:<br>Quote: *"In the group I felt a sense of acceptance, of being more among others who understand and therefore don't expect more of me than I can do. Especially the reassurance that I am part of a very diverse and normal group of people who have in common the loss of a loved one by suicide. The reassurance that I am still a good - OK - person, not a pariah. Our common experience of helplessness before another suicide allows me to believe that I am not to blame - none of us are - and that I can go on with my life"*. (p253)<br><br>Increased sense of self-worth by reaching out to each other (p 253):<br>Quote: *I myself feel better when I think I'm helping someone else - usually a person whose bereavement is more recent than mine"* p253<br><br>Gain a sense of control over their lives by developing coping strategies (p253)		
Feigelman et al. 2008 (USA)	**MOTIVATION:**<br>Why did you seek your Internet support group? (N = 200)<br>Percent endorsing this<br>1. The Internet group is consistent with my personality needs, where having 24/7 availability is needed (64%).<br>2. I like the democratic participatory style of the Internet group (38%).<br>3. I like the structured leadership of the Internet group (34%).<br>4. Leadership does a good job harmonizing the diverse elements within our Internet support group (34%).<br>5. I have no other alternative; there are no face-to-face groups within easy traveling distance (30%).<br>6. I am "turned off" by local face-to-face groups (25%).<br><br>**IMPACT:**<br>Open ended questions - what was the internet support group helpful for:<br>A) *"I honestly don't know how I would have made it through the months following my son's departure if this Internet support group hadn't existed."*<br>B) *"The Internet group has been there when no one else was or cared. POS is the main reason I am alive today."*<br>C) *"Without the support that I received I would be dead from my own suicide, I have*	The privacy in the internet group, 24/7 because they had a help source whenever they needed it (p 228)<br><br>Good leadership i.e. democratic; structured and managing group diversity well.<br><br>Format: Internet forum more desirable than face-to-face format for some participants.	Authors: Hindering (healing in the support group) responses fell into several different (and sometimes contradictory) types: for some the group was perceived as having an overly Christian faith bias; for others it was felt to be insufficiently spiritual; some complained of too much repetition to the subjects discussed, while others felt they were overwhelmed by the high volume of different items that were never fully processed. Another common complaint was finding it difficult to get responses to one's postings. Others complained that the support group increased their

	suffered from depression."<br>D) *"The group helped save my life literally as well as figuratively."*<br>E) *"POS helped me more than mere words can say. I can't even think about how I would be today, if it wasn't for this group"*. (p229)<br><br>From discussion:<br>Internet support group as a life safer: Only six respondents remarked that the Internet support group participation contributed to worsening their feelings of depression, while 14 others commented that they perceived this group as their lifesaver, helping them to stay alive after having come to their lowest point of depression and despair. With 80% of support group members describing themselves as presently depressed, and 35% harbouring suicide thoughts sometimes or more often during the past year, the significance of these lifesaving remarks takes on additional meaning. p240		feelings of depression and sadness, as they dealt with the comments of others who appeared to be stuck in their grief or suffering from multiple suicide losses. (p 229/230)
Feigelman & Feigelman, 2008 (USA)	**IMPACT:**<br>"All-in-the-Same Boat" Phenomenon:<br>Less isolation through sharing feelings and experiences (e.g. stigma, confusion inability to perform daily activities and loss of one's belief system (p 292)<br>Quote: *"I don't feel guilty about Pat's (son) death now and coming to group has been a huge help for me. In the beginning it was the only safe place to speak about my son"*. P292<br><br>Discussing a Taboo Area<br>The group provides a safe place where group members feel free to express taboo feelings or address taboo subjects. This gives the members a sense of relief and diminishes the sense of shame, guilt and isolation due to keeping feelings and subjects hidden (p.292)<br>Quote: "*The night my son hanged himself, Jeff had been coming down from a cocaine binge. I was ashamed to talk about this here before because I wanted to protect him and me. I was afraid that you would think less of him—his life, his death, and my loss because of his cocaine use. I know how society looks at people who use drugs. Jeff was very talented and loving and he deserves more than that"*. P293<br><br>Mutual support: Authors: Acceptance, offer hope for decrease of the pain, suggest	That the participants had different perspectives and engage in a dialogue where they shared different understanding e.g. reasoned in different kinship or a another "stage" in the process of healing/grieving	Stated in the conclusion that some people withdraw reasoned in: displeasure with the facilitator and other group members for monopolizing the group's meeting time; feeling that the group was overly preoccupied with issues peripheral to recovery after suicide; sharing the belief that continued involvement with the group "brought them down." (p 300)

	concrete ways to cope with problems, let go of guilt and blameworthiness (p 293)<br>Quote: "*When we first started coming here eleven months ago, we felt hopeless and even though we have lots of friends and family, this place felt different—a room full of strangers and yet we felt comfortable. People really understood us. In the beginning, I was amazed to see how some people, here longer than us, were functioning so well, even laughing a little. Now, I meet new people coming here for the first time and I can see that Robin and I are not in the same place. Our pain feels different. Time does that. Someone once said these meetings work because of talk, tears, and time. I think that's true*". p293<br>Quote: "*After my son's death I felt guilty and ashamed. After all, there had been warning signs and I was a trained mental health professional….Over time, while helping others (including other mental health professionals who were also survivors) to feel less responsible for a death they could not have prevented, I began to be less hard on myself. My son had made the decision to end his life in spite of everything I had done or could have done*". p294<br><br>Problem solving:<br>Opportunity to problem solve with the help of other members who were in similar situations and offer different perspectives and ways of looking at the problem. (p 294/5)<br><br>Sharing data:<br>Share experiences of healing and coping which helps others. E.g. ask others if something they feel is normal for the grief/healing process, dealing with other people, activities that help etc. (p 295)<br>Quote: *"This second year without my son John feels even harder. People expect me to "move on" and to get rid of John's clothing and possessions, but I can't. Is this normal? Will I ever be able to clean out the closet? Do I have to?"* p295<br><br>Dialectical processes where someone with a different kinship relationship to deceased or another experience can say something making each other reflect on views and beliefs (P296)<br><br>Mutual demand: Making suggestions to motivate other members with the		

	struggles that are often universal in the survivor group - such as the conflict between going on with life and feeling guilty. In doing so they empower each other to grow and change, and move from victim to survivor (p 297).<br><br>Rehearsal: discussion of how to deal with people, situations, special days etc. which can reduce anticipatory anxiety. (p 297/8)<br><br>Universal Perspective: understanding suicide from a social/institutional perspective (e.g. failure of MHS) allows members to move way from personal blame to social action. (p 298)<br><br>The "Strength-in-Numbers" Phenomenon: unite with other suicide survivors to initiate social change (p 299) (This do not seem to be based on the analysis of data).		
Barlow et al. 2010 (Canada)	**MOTIVATION:**<br>To get the support of someone who could ''understand my situation"; to share coping strategies; offer hope; provide answers; keep them stable; increase their understanding of the suicide death; and provide closure. P 920/921<br><br>**IMPACT:**<br>QUATNITATIVE RESULTS<br>-Peer support was rated 4.3 and 4.5 out of 5 by peer supporters and clients respectfully.<br>-Comfort was rated an average of 4.7 out of 5 (both together)<br>-Helpfulness was rated average of 4.4 out of 5 (both together).<br><br>Hogan's Grief Reaction Checklist was administered to all participants pre and post intervention and showed decreased despair, panic, blame and anger, detachment, and disorganisation; and increased personal growth. Three out of six items showed statistically significant differences ($p<0.05$): despair, detachment, and disorganization. Cohen's ds revealed that effect sizes were large for despair and disorganization (.84 and 1.10) and medium sized for detachment (0.72).<br><br>QUALITATIVE RESULTS	Authors: Matches based on similar losses were a powerful factor in their ability to connect with each other (p 924)<br><br>Authors: The participants noted the following considerations of the peer supporters that contributed to the program's effectiveness: willingness to accommodate the participants' schedules, flexibility of meeting place, meetings in neutral areas, additional support via e-mail and telephone, and ability to be fully present.<br><br>Authors: Peer supporters felt that monitoring and reflecting on client progress and their	Logistics was an issue in terms of finding appropriate meeting places (safety and privacy) and setting up meeting times because of the time constraints of the participants, but no results about it hindering outcomes.<br><br>Authors: Recurring themes among the peer supporters included a sense of vulnerability in terms of how to assess their effectiveness; frustration if their partner was being unresponsive to their overtures of assistance; uncertainty about what were appropriate boundaries and how to set and maintain them,

	CLIENTS:<br>Authors: All clients believed that talking about emerging extended family matters was a particularly supportive aspect of the meetings. In addition, they noted that expressing feelings of frustration and anger, talking about the deceased, talking to someone with a similar experience, sharing coping strategies, and receiving information on the grieving process and available resources were helpful. p921<br><br>Authors: Clients reported that the meetings were helpful because they felt listened to by a person with a similar experience, which then contributed to new levels of understanding about suicide and themselves. On a pragmatic level, the clients appreciated peer supporter suggestions and their knowledge of resources as well as their willingness to engage in problem solving, which often led to positive action steps. p922<br><br>Hope:<br>Several clients noted that the peer supported offered hope - *''a light at the end of the tunnel"*. p922<br><br>ON HOW GROUP CONTRIBUTED TO HEALING:<br>Enhanced well-being:<br>Quote: *''I felt good after the meetings. I'm in a better place now than I was back then.'' ''Because I'm feeling much better—about myself.''* p924<br><br>Emotional expression in a one-to-one setting:<br>Quote: *''It was good to see his [the volunteer's] pain. Ten years later he still had a tear in his eye.''* p924<br><br>Normalizing of the loss experience:<br>Quote: *''It is normal—that's how you feel—this is what you can expect to feel down the road - several years out of it – you know—and you can see that it is better—but it's not going to go away—seeing someone at a different stage in their life"* p924<br><br>Memorializing of the deceased:	relationship with the client provided assurance that ''I was doing a good job'' was evidence of forward movement in the client, offered a respite from worry about the client, and built confidence in their ability to establish and maintain a trusting relationship with the client. p921<br><br>For peer supporters, meetings (monthly debriefing and educational sessions), the guidance and support offered by CMHA counsellors and the lack of 'rules to follow' were cited as helpful.	dissatisfaction with the management of peer matches that were proving unsuccessful, coupled with uncertainty about how to terminate such relationships.

	Quote: *''Seeing it still so fresh [in the volunteer] was reassuring because you don't want to forget.''* p924<br><br>Connection:<br>Quote: *''Nice to talk to somebody who had gone through the same thing.''* p924<br><br>ACCEPTABILITY:<br>In response to what was missing from the study, clients typically reported, *''Nothing was missing.''* However, one client declared that the meetings were too long.<br><br>PEER SUPPORTERS:<br>Authors: On what was helpful during their meetings, peer supporters believed that noticing themes, informing clients of coping strategies, asking questions that assisted the client to open up, and reviewing the value of the meeting all were helpful for the client. In addition, peer supporters reported that sharing their personal experience with grief, observing client progress, talking about family concerns, and receiving feedback from the client on the helpfulness of their meeting facilitated healing. p921<br><br>Peer supporters assessed that talking about emotions and developing insights about themselves and the client were mutually therapeutic. p922<br><br>ON HOW GROUP CONTRIBUTED TO HEALING:<br>Cognitive restructuring:<br>Quote: *''I started thinking about things a little differently.''* p924<br><br>Connection:<br>Quote: *''Every opportunity you get to be with other people, talk about things, it just helps and makes it easier.''* p924<br><br>Invitation to personal growth and self-reflection:<br>Quote: *''It contributes to more growth, more growth personally and to do with my*		

	grief.''/''It was a reminder of where I've been and how far along I've come.''/''It helped me re-evaluate my life even when I thought I recovered.'' p924<br><br>Memorializing of the deceased:<br>Quote: *''How could I explain—introspection and all this—records were not erased.''* p924<br><br>Satisfaction:<br>Quote: *"I see this person being more whole again. I'm giving back. It helped to be of service.''* p924<br><br>Contributed to meaning making about the suicide:<br>Quote: *''I was making sense of it by helping others.''* p924<br><br>Giving back:<br>Quote: *''It evens things up. Helping another when possibly you were unable to help [the deceased].''* p924		
Feigelman & Feigelman, 2011 (US)	**IMPACT:**<br>Reduce self-blame:<br>Quote: *"But the support group. That was wonderful! It felt so good to know others in the same boat as me. I was not the only one losing a husband to a suicide. In the group I learned to stop blaming myself. There was another widow in our group whose husband shot himself right in front of her in their living room. If she could stop blaming herself for her husband's death, then there was every incentive for me to do the same. The support group showed me that I was not the only one with my problems and I absolutely must stop torturing myself" p169*<br><br>Process grief and rebuild confidence:<br>Quote: *"Hearing other survivors' loss stories helped me to relive mine. I had to come to terms with my loss and the support group helped me do that. I learned a great deal from it. Part of my problem after my son died was not having much confidence in myself. I was shattered after Jackie died. I kept blaming myself; he was so young. Working through the grief helped me to rebuild my self-confidence. Eventually I got back enough nerve to return to high school science teaching. It took me about 5 years to do that and I think I owe it to*	Good facilitator skills e.g. ability to control group and thus prevent members for monopolising group; encouraging group members to participate; preventing monopolisation by some members; responding to attendees' non-verbal cues; remaining silent and allowing group members to fill the silence; identifying members that could help each other and putting them in contact with each other.<br><br>One cornerstone of the support	This article is focussed on people leaving the group and thus may have had more to say about elements that hindered outcomes.<br><br>Bad facilitation in terms of not easing new members in, allowing some members to monopolise conversation and not facilitating the group to move through topics rather than going over the same issues week after week.<br><br>The issue of group monopolization was raised

	the groups to helping me". p178<br><br>Long term bereaved who had left a support group explained that they had gained from being able to speak freely and openly, and from the ongoing support they received from people in the group who had become friends.<br>Quote: *"wonderful medicine; I was free to speak my mind and I knew I would be understood. I felt safe there. There wasn't anything I couldn't talk about in the group. We're all in that same place together"* 178<br>Quote: *"They [her support group friends] have been like a family to me when my own family failed and didn't know what to do or say to us; they still avoid mentioning our deceased son and act as if he had been consumed by the plague or something"*. p180<br><br>Evidence of Posttraumatic growth among long term bereaved:<br>Authors: Post-traumatic growth is reflected in social change actions, when survivors act collectively to alter the fabric of society, to prevent suicide, and to diminish the stigma that now surrounds it and other mental health problems. Examples given on pages 179-181.<br><br>Support group benefits (from conclusion):<br>-Some participants said *"they don't know have they would have made it through without the peer support group"*.<br>-It made them feel understood in their new and difficult roles as bereaved by suicide.<br>-They found role models whose resilience inspired them and gave them hope<br>-They could measure their own progress in healing by listening to other's stories<br>-They grew in terms of accepting their loss and feeling able to move on from the group<br>-They gained friendship and support networks (p 182)	groups' success is the permission it offers survivors to talk about ALL facets of their loss, including some themes viewed as taboo by non-survivors.<br><br>Right timing is important – even though it is individual:<br>Authors: It is sometimes suggested that survivors should not begin attending support groups until a certain amount of time has passed since the loss. Yet, there appears to be no hard and fast rule on this. We have seen some survivors benefit from participation as recently as 1 week after the death of a loved one. And we have seen others, starting their participation more than a year after a loss, who are still not entirely comfortable and question the value of support group participation. The time interval depends entirely upon the individual survivor and his capacity to feel sufficiently safe, supported, and in a comfortable place and ready to share and exchange loss stories with other survivors. P 173/174	several times while the formation of cliques within groups was also cited as a barrier to participation:<br>Quote: *"I was put off by some other members who usually dominated discussions. If I could get a comment in between these five big talkers I was very lucky. After meetings, this same little group and the facilitator usually went out to a diner for coffee"*. p175<br><br>Some members felt retraumatized and down as a result of listening to other people's stories of loss:<br>Quote: *"Maybe I made a mistake by starting here too soon after my daughter's death. Maybe we should have waited longer before we started. I just couldn't sit still during the meeting, hearing about others' tragic losses. It was too much for me and it brought me right back to the scene of my daughter's death.... It was much too distressing. I think my husband and I will try going to a general bereavement support group where it might be easier for us now"*. p174

			Quote: *"I was in the group for 7 years; it was enough already. Hearing the new people come in and tell their loss stories—seeing their tears, seeing them all welled up with so much emotion, guilt, and shock—it all brought me down and back to the place I started from. I didn't need this anymore"*. p179<br><br>While some members sought social connections from their group, others felt that social obligations were burdensome. Quote: *"I declined invitations to attend social events that the facilitator or others organized, like summer picnics and occasional dinner parties. However, when I saw the people again at later meetings I had the feeling that some of them thought I was stuck up and unfriendly. I resented all these additional social obligations beyond the meetings. I preferred spending most of my free time with my family and friends"* p176.<br><br>Support groups must fit into the framework of the participants' everyday life for

			them to be able to partake. E.g. transportation, other family member's needs (p 177)<br><br>Too many members in group, thus it lacked the intimacy of smaller groups (p175).
Marijke Schotanus-Dijktra et al. 2013 (Belgium and Netherlands)	Positive about online support (Messages containing positive experiences or information about the online forum for the bereaved by suicide) (9%):<br>Quote: *"I am also very happy that I can read other people's experiences on this forum."* p31		Negative about online support (Messages containing negative experiences or information about the online forum for the bereaved by suicide) (1%):<br>Quote: *"I am not often on this forum, I'm afraid it all gets to me too much."* p31
Bailey et al. 2017 (UK, USA, Canada and Australia)	**IMPACT:**<br>**Benefits with more than 50 % agreement:**<br>-I like the 24/7 availability of the forum<br>-Using the forum makes me feel less alone<br>-The forum helps me to cope with the pain and sadness of suicide bereavement<br>-I like talking with other people who have been through the same thing that I have<br>-The forum helps me to get through difficult times, such as anniversaries and birthdays<br>-I find it beneficial to help others struggling with suicide bereavement issues<br>-Talking with others on the forum helps (or has helped) me find ways to talk about suicide openly<br>-The forum allows me to preserve the memory of the person(s) I know who died by suicide<br>-Talking with others on the forum helps (or has helped) me with family difficulties that happened after the person died<br>-I use the forum to find out about suicide prevention and bereavement resources<br>-I like that I can choose to remain anonymous on the forum<br>-Talking with others on the forum helps (or has helped) me to deal with rejection	24/7 availability<br>Anonymity	Some participants agreed that the moderator did not do enough to help members who were upset (5.4%) or to remove upsetting posts (2.7%). 4.5 % reported they had been exposed to inaccurate information and 21 (9.5%) agreed that internet trolls posted on the forum. (p398).

	by family members, friends, co-workers and/or neighbours<br>-Talking with others on the forum helps (or has helped) me to decide what to tell others (such as children, other family members, friends) about suicide (p 398)<br><br>**NEGATIVE IMPACTS** of using the forums were reported including becoming upset after reading something on the forum (19.4%), becoming upset after talking with others about experiences (14%), becoming depressed (5.4%), and feeling suicidal (1.8%). Eighteen (8.1%) reported they had spent so much time on the forum that they had stopped doing other things, while 20 (9%) agreed that the forum made it harder to move on with their life. 7.2% reported that they did not get the support they needed from other members.		
Silvén Hagström, 2017 (Sweden)	**MOTIVATION:**<br>Authors: The mourners of parental suicide recurrently describe their participation in the chat blog as originating from feelings of loneliness and Otherness in their ordinary social networks. This common circumstance for communication affects the kind of support they are looking for in this context (p 782)<br><br>**IMPACT:**<br>Mutual recognition even where the kinship relationship to the deceased differs:<br>Authors: This means for example that a daughter who lost her father to suicide can, at least to some extent, recognize herself in a mother's writing about her grief following her son's suicide. Consequently, all the bereaved can get something out of reading the experiences of others (p 782)<br>Quote: *"Hi! I have read your website and I recognize myself in many things! I lost my dad by suicide in 2006. My whole world fell apart. I never had the time to reconcile with him! And that hurts so much. I dream of him and wake up in a panic! But continue what you are doing! You do a great job. You can contact me!" p782*<br><br>Need to construct meaning in the parent's suicide:<br>Quote: *"I am happy that I found this website. I don't know where to turn. It's now about four weeks since my father killed himself and I hate the fact that I will spend the rest of my life wondering why. He was almost always happy, had recently started a new job and registered for a marathon. He didn't leave any warning or explanation. I always called*		

	myself 'daddy's girl' and now I don't have a dad anymore" P783.<br><br>Authors: The Internet arena for social support offers the opportunity to communicate not only with other bereaved, but also with the deceased parent in what has been described as a 'continued bond', which can work as a resource in the children's meaning construction (p 784)<br><br>Authors: Taken together, the voices of the parentally bereaved participants highlight the importance of a social context for support that allows the meanings of suicide to be negotiated, and where new understandings of suicide can be introduced that resist these stigmatizing discourses. (p785)<br><br>New opportunities for meaning making: Editors and other survivors counter children's interpretation of their parent's suicide as selfish - instead urging them to reframe their thinking on suicide as an involuntary act and to seek reasons outside of blaming the deceased or themselves. However, there is no information on how this reframing is experienced.<br>Quote: *"I think your mother must have been feeling very bad when she died. Otherwise, you don't leave your small children. I think you need to ask your father ALL your uncomfortable questions!! [...] If people tell you that she chose to kill herself I urge you to read the tabs on this website!! No one wants to die. It's the pain. You can't cope any longer"* p786		
Ali & Lucock, 2020 (UK)	**MOTIVATION:**<br>Those bereaved by suicide wanted to meet others bereaved by suicide to make sense of the suicide, to get support with coping, and to be in a safe non-judgmental with people with knowledge of suicide bereavement and where there was no stigma.<br><br>**IMPACT:**<br>Helped participants to process grief:<br>Quote: *"I found SOBS and the Compassionate Friends support people who've lost a child in any way. Sharing helps quite a lot with your grief, because you can talk about it and they can talk about theirs and you swap bits of information and feelings."* (Mother) (p54)	Finding people with same gender and kinship to the deceased, which provides a better mutual understanding. For example, although there were few men in the group, two that had lost wives bonded:<br>Quote: *"I know why he's feeling like that and it's something between us two that we can talk about that the other group can't" (p54).*	Group format – discomfort speaking in group:<br>Quote: *"I've had people who've phoned me that say I couldn't come to a group and talk in a room full of strangers" (p55).*<br><br>A minority of participants felt unable to empathise with the majority of those attending SOBS, because of a different

	Helped participants gain a greater understanding of their own and others' experiences through the process of sharing and hearing others' stories. This helped many participants to put their experience into perspective:<br>Quote: *"I think I'm in a bad situation, but then I listen to other people then I think "you're in a worse situation". It does open your eyes to others". (p54)*<br><br>Facilitated acceptance that they could not take responsibility for the death and this reduced some of the guilt they felt.<br>No quote provided for this.<br><br>Mutual understanding and empathy:<br>Quote: *"I say at SOBS you can laugh about things, where in a group away from SOBS…I suppose if you're in a group of people, say the hospital or something like that with professionals, if you laughed they'd all look at you like that, what's wrong with this person are they having a nervous breakdown? […] but sometimes the laugh is a relief because you know somebody's understanding what you're talking about"*. (p54)<br><br>Provided hope:<br>Quote: "*The value of the group is that you can see other people are further down the road of coping and it gives you the hope that you can cope too"*. (p55)<br><br>A changed sense of selves and identity which was empowering:<br>Quote: *"Participants described a changed sense of self and identity and defined themselves as 'survivors' – "because we are "survivors of bereavement" not "victims" as such." (p55)*<br><br>Sense of belonging and friendships.<br>The group did shared activities outside of the group.<br>No quote.	To meet people that are longer in their grief:<br>Authors: Longer-term regulars helped others, became role models and provided hope for new attendees rather than seeking support themselves. (p 55)	kinship relationship with the deceased.<br>No quote provided in text<br><br>Another difficulty for a few participants was finding others in the group who had had an estranged or distant relationship with the deceased, because their grief experiences felt different compared with the majority of the group who had a closer relationship with the deceased. (p 55)<br><br>Geographical distance and committing to the dates and times.
Tosini & Fraccaro, 2020 (Italy)	**Impact**<br>Fostered a sense of belonging and connectedness which contrasted with their feeling of social isolation, due to stigmatization and self-stigmatization:<br>Quote: "*Certain things, certain emotions, you don't feel like talking about them even to*	Mixed membership of those who had attempted suicide and suicide bereaved:<br>Suicide survivors better	

	the closest relatives because you feel … that they can't understand you, because anyhow they haven't lived through the same experience […] you try to speak to people [in the support group] who have experienced the same occurrence, the same situation, because you know that perhaps help can come from there" (p5)<br><br>Felt specifically more connected with suicide support group as opposed to support groups for all types of grief:<br>Quote: *In the group [dedicated to all types of grief], the people welcomed me […] Everyone always had a word of comfort for me. I think that losing someone, however – I lost my father of a natural death – is something different […]. And so I attend this group and I feel good here, but… it's not so specific. And so quite a few times I talk, and they don't understand me […] If I could attend [the group for suicide survivors], it would be different" (p5)*<br><br>Benefit of sharing their emotions and interpretations:<br>Quote: *"the group meetings were more useful than psychotherapy... to have so many points of views together... yes, you have more hues […] There is true emotional closeness […] The group is my space: it allows me to release this thing [i.e., her trauma], to process it, because otherwise I suppress it."* (p5)<br><br>Source of solidarity and crucial handholds that helped survivors "climb" their personal "glass wall". Overcoming conflicting feelings and sense of guilt by providing new perspectives and strategies:<br>Quote: *"the group is welcoming: you leave here a little bit of your pain. You take something that helps you from some members of the group. Each one of us has inner resources"* (p5)	understood the desperation of those who attempted suicide and this lessened their anger and sense of abandonment at their own loss. Similarly, the suicide survivors felt a diminished sense of guilt insofar as they learned that people who attempt suicide often believe they are beyond help, and therefore hide their suffering and suicidal thoughts	
